# Data linkage infrastructure for cross-jurisdictional health-related research in Australia

**DOI:** 10.1186/1472-6963-12-480

**Published:** 2012-12-29

**Authors:** James H Boyd, Anna M Ferrante, Christine M O’Keefe, Alfred J Bass, Sean M Randall, James B Semmens

**Affiliations:** 1 Curtin University, Perth, Western Australia; 2 CSIRO Mathematics, Informatics and Statistics, Canberra, ACT, Australia; 3 Menzies Research Institute, Tasmania, Australia

**Keywords:** Data linkage, Infrastructure, Population, Health, Research

## Abstract

**Background:**

The Centre for Data Linkage (CDL) has been established to enable national and cross-jurisdictional health-related research in Australia. It has been funded through the Population Health Research Network (PHRN), a national initiative established under the National Collaborative Research Infrastructure Strategy (NCRIS). This paper describes the development of the processes and methodology required to create cross-jurisdictional research infrastructure and enable aggregation of State and Territory linkages into a single linkage “map”.

**Methods:**

The CDL has implemented a linkage model which incorporates best practice in data linkage and adheres to data integration principles set down by the Australian Government. Working closely with data custodians and State-based data linkage facilities, the CDL has designed and implemented a linkage system to enable research at national or cross-jurisdictional level. A secure operational environment has also been established with strong governance arrangements to maximise privacy and the confidentiality of data.

**Results:**

The development and implementation of a cross-jurisdictional linkage model overcomes a number of challenges associated with the federated nature of health data collections in Australia. The infrastructure expands Australia’s data linkage capability and provides opportunities for population-level research. The CDL linkage model, infrastructure architecture and governance arrangements are presented. The quality and capability of the new infrastructure is demonstrated through the conduct of data linkage for the first PHRN Proof of Concept Collaboration project, where more than 25 million records were successfully linked to a very high quality.

**Conclusions:**

This infrastructure provides researchers and policy-makers with the ability to undertake linkage-based research that extends across jurisdictional boundaries. It represents an advance in Australia’s national data linkage capabilities and sets the scene for stronger government-research collaboration.

## Background

### Benefits of data linkage to research, policy making and service delivery

Administrative datasets constitute a significant information resource for government and are used to manage, monitor, assess and review a range of service areas. They are also used in research to provide insight into significant health issues, to support health policy development and improve clinical practice and service delivery. Additional value can be obtained from these administrative collections through data linkage. This process allows data from different sources, including disease registers and clinical datasets, to be brought together to provide richer information. The benefits of linked data include reduced data collection costs and more detailed and extensive analysis [1-6].

### Data linkage infrastructure developments

Despite recognition of the value of data linkage by government and the research community, dedicated infrastructure to sustain and support data linkage activity is limited. Data linkage “systems” or “facilities” exist in only a handful of countries including Canada [7], England (Oxford) [8], Scotland [9], Australia [10] and most recently in Wales through the development of the SAIL system [11]. These production-level systems undertake linkage on a routine basis, servicing the statistical and research needs of both government and University researchers.

In Australia, purpose-built data linkage infrastructure was first established in 1995 in Western Australia. The Western Australia Data Linkage System (WADLS) emerged from a collaboration between the University of Western Australia’s School of Population Health and the Western Australia (WA) Department of Health. WADLS comprises a complex probabilistic data matching system to create, store, update and retrieve links between over 40 population-based administrative and research health data collections in WA [12]. Following the success of the WADLS and in recognition of the power of the resulting linked research data, the Centre for Health Record Linkage (CHeReL) was established in 2006 in New South Wales (NSW) to undertake data linkage for NSW and the Australian Capital Territory [13]. Hosted by the NSW Cancer Institute, CHeReL is a joint venture between eight institutions. It has developed quickly to incorporate the routine linkage of a number of strategic, core datasets.

### PHRN initiative

Further investment in Australia’s data linkage capability occurred in 2006 when the Australian government allocated $20 million to further develop data linkage infrastructure under the National Collaborative Research Infrastructure Strategy (NCRIS). State and Territory governments and academic partners invested a further $32 million to support the capability. The initiative, known as the Population Health Research Network (PHRN), included the establishment of data linkage units in all other Australian States, the formation of the Centre for Data Linkage (CDL) for national or cross-jurisdictional linkage, the development of a secure remote access laboratory for researchers, and a data delivery system for the secure electronic transfer of data between PHRN participants and relevant stakeholders. The purpose of the PHRN is to “provide researchers in Australia with the capability to link de-identified data from a diverse and rich range of health datasets, across jurisdictions and sectors, to carry out nationally and internationally significant population-level research, to improve health and wellbeing and enhance the effectiveness and efficiency of health services” [14].

A core component of the PHRN infrastructure has been the development of national or “cross-jurisdictional” linkage capability i.e. the ability to link data from more than one State or Territory. Given the federated nature of health care service delivery in Australia (i.e. some services are delivered and administered at State level, while others are delivered and administered at a national or “Commonwealth” level), cross-jurisdictional linkage is an essential component of national infrastructure. Without cross-jurisdictional data linkage capabilities, research aimed at national level or targeting issues of common interest (e.g. health service use along border areas) cannot be undertaken. The remainder of this paper describes the development of the processes and data linkage methodology required to create a cross-jurisdictional research infrastructure and the aggregation of State and Territory linkages into a single system.

## Methods

Under the PHRN initiative, the CDL was tasked with “establishing a secure and efficient data linkage system to facilitate linkage between jurisdictional datasets, and between these datasets and research datasets using demographic data” [14]. To fulfil this function, the CDL engaged in the:

i) Development of a cross-jurisdictional operational model

ii) Specification and implementation of a secure IT environment including linkage software; and

iii) Development and adoption of strong governance arrangements

### CDL operational model development

**Figure 1:**
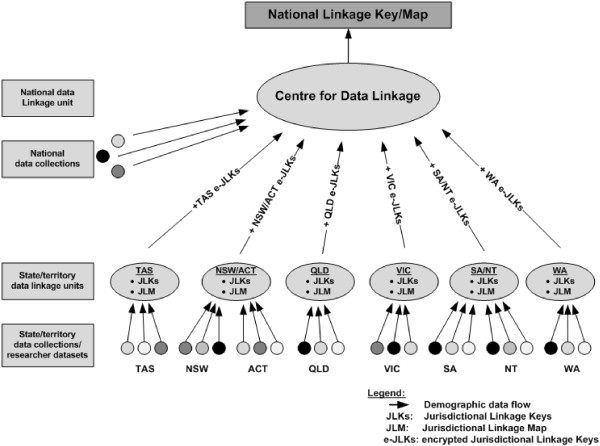
Layered Linkage Model of the PHRN.

The operations and infrastructure in the CDL build on the models created in both WADLS and CHeReL. The Cross-Jurisdictional Operational Model was developed in wide and open consultation with PHRN members and related stakeholders [15]. The Model incorporates a separated and layered linkage approach where State/Territory linkages are conducted by individual State-based or “jurisdictional” linkage units, while cross-jurisdictional or “national” linkages are conducted by the CDL (see Figure 1).

This layered model maximises the skills and experience in data linkage across Australia and builds on the success of well established data linkage units in WA and NSW/ACT. It involves a multi-tier operating structure with standardised governance arrangements which are responsive to researchers needs. The state/territory data linkage units have had a major influenced on the development of the model and the CDL has benefited from working with state/territory data linkage units to understand the data, the technologies and researcher needs. The layered model also allows efficient control over aspects such as skill development, resource utilisation, operational efficiency and the application of standards across data linkage units.

A best practice ‘separation’ principle operates in the Model at both State (or “jurisdictional”) and CDL levels [16]. Under this principle, the process of data linkage (and the data items used in linkage activity) is kept separate from the processes that extract and deliver content or clinical data for researchers. Data flows for cross-jurisdictional linkage comprise three distinct phases:

•Flow of data for linkage

•Provision of project specific linkage keys

•Extraction of research data

Phase One of the data flow model is about **the linkage process**. The data used for linkage involves only a limited set of variables, typically demographic data (e.g. name, date of birth, address, date of event). This information is used for linkage purposes only. A Data Custodian provides demographic data and related record identifiers to the Jurisdictional Data Linkage Unit. The Jurisdictional Linkage Unit uses this data to undertake state-based linkages for state-based research projects. For cross-jurisdictional projects, the local Linkage Unit provides the demographic data and encrypted record identifiers to the CDL. The CDL uses this information to link data across multiple jurisdictions.

An important element of the Cross-Jurisdictional Model is the creation and maintenance of a National Linkage Map [17]. Following the linkage process, the CDL assigns the same reference key – a National Linkage Key (NLK) - to each record that is considered to belong to the same person. The reference between the Unique Record Identifier (RecIDs) of each record and the NLK creates the national linkage map (i.e. a direct list showing the national linkage key corresponding to each unique record identifier). Allocation of the NLKs allows the system to group records within the National Linkage Map to show which sets of entries are considered to refer to the same person.

Each NLK only has value within the context of the National Linkage Map, which associates them with pointers to health records. The Unique Record Identifiers contained in the Map are encrypted and each is used as a pointer to the information held by data providers. It is important to note that the National Linkage Map does not contain any demographic or content variables. When extracted, information from the National Linkage Map are masked and then encrypted before being supplied to Data Custodians for approved research projects. Phase Two of the process is the **provision of project-specific linkage keys** which enables research datasets to be extracted and merged anonymously by researchers. For each cross-jurisdictional project, the CDL returns to the local Jurisdictional Linkage Unit a file with the record identifiers and project-specific linkage keys. Each project is issued with a unique set of project-specific linkage keys. The local Linkage Unit passes the project-specific key and record identifiers to the Data Custodian who then proceeds to the final phase of the process (data extraction).

Phase Three, **extraction of research data** for approved projects, takes place only after Phase One and Phase Two have been completed. For each cross-jurisdictional research project, content data is extracted by the Data Custodian. It consists of project-specific linkage keys and only those variables which the researcher has been authorized to access. The dataset does not contain any identifying data items (e.g. name). The linkage keys in the dataset are project-specific so that researchers cannot collude and bring together data from different projects. Once the researcher is provided with data from all relevant Data Custodians, records can be merged using the project-specific linkage key and then used in analyses.

**Figure 2:**
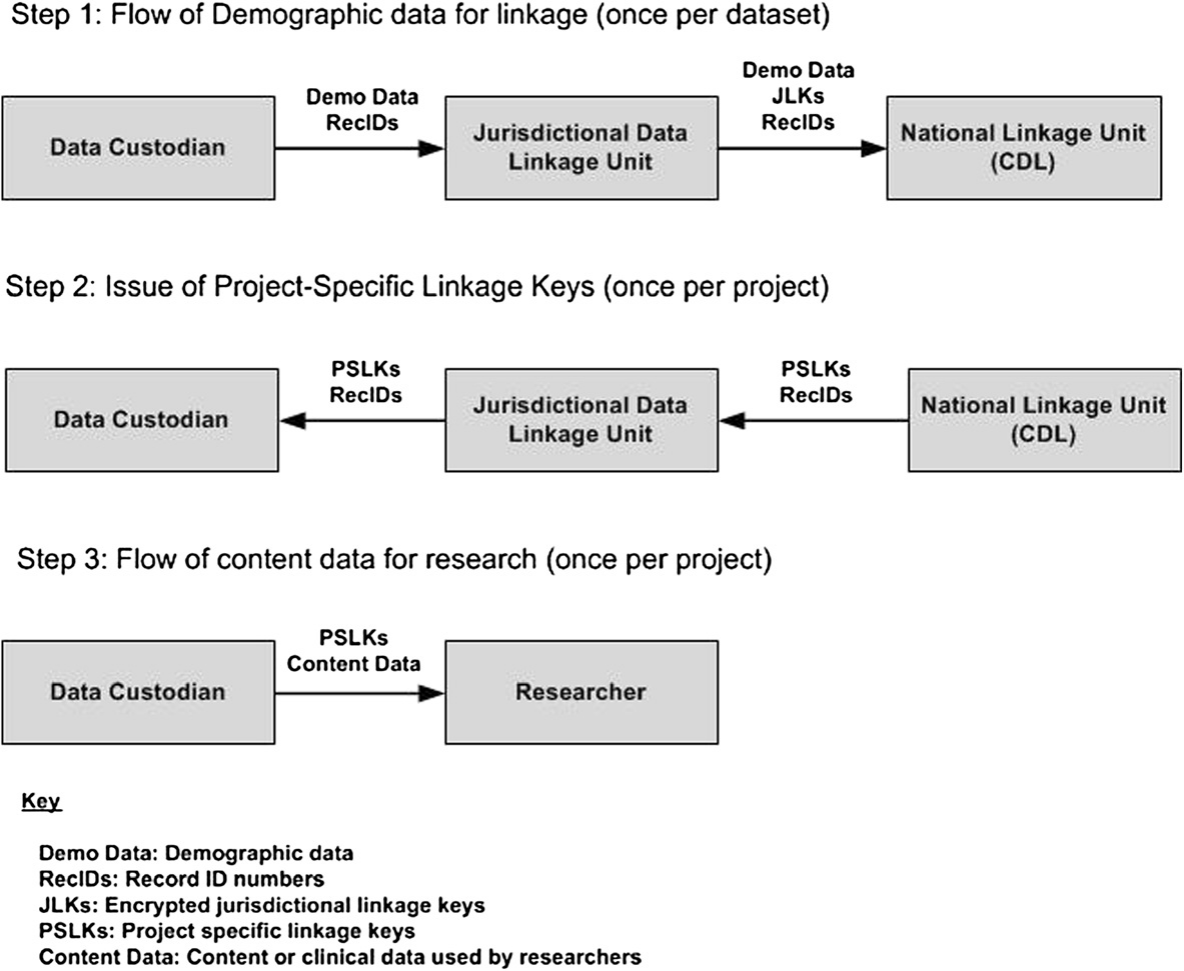
Cross-jurisdictional data flows.

As Figure 2 shows, the Data Custodian is an integral part of all steps of the process and directly controls access to their data. This Model does not involve a central data repository which means that custodians only release data on a project by project basis. The CDL does not hold clinical or content data, but links the demographic data that has been separated from the remainder of each dataset to create ‘linkage keys’. Clinical or service information is not needed by the CDL and is not provided to it and the researcher receives only that part of the record that they have approval to see (without any demographic or identifying information).

With the model separating the linkage and research data and functions, access to reliable metadata during the linkage and analytical part of each cross jurisdictional research project is important. In Australia the METEoR system is one such metadata repository that provides a single-source dataset of definitions (including those administrative in nature) at a national level. This will be a useful resource to align the definitions across jurisdictional datasets*.*

### Secure IT environment

To implement the Operational Model, the IT infrastructure arrangements for CDL had to provide a secure controlled environment for working with name-identified data. Understanding the sensitive nature of identifying information assets, the CDL designed its operations to accommodate datasets from State and Commonwealth organisations whilst applying the highest level of security. As well as ensuring that identifying demographic information was handled separately from any content or clinical data as part of its data flows, the CDL established a secure IT infrastructure to protect these information assets throughout the process.

**Figure 3:**
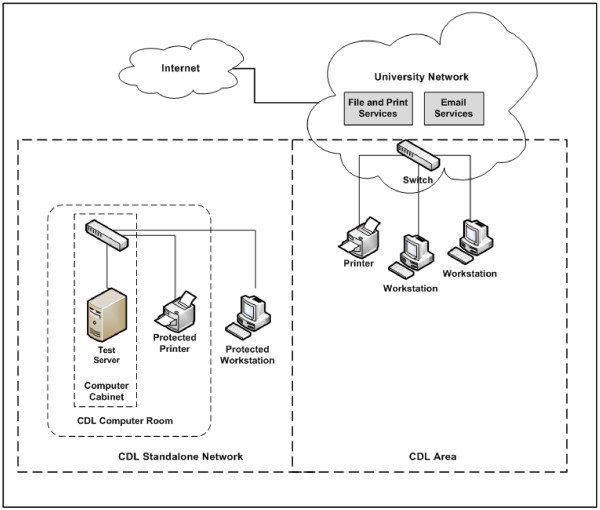
**CDL IT infrastructure configuration.** * For Proof of Concept, computing capabilities were enhanced; however, the basic configuration of IT infrastructure was unchanged. Additional computing resources included two new servers and UPS.

A secure stand-alone network (the CDL stand-alone network) was designed in consultation with the PHRN to enable the storage and processing of demographic data received from the jurisdictional linkage units, researchers and other sources. The Australian Department of Defence publication ACSI 33 Australian Government ICT Security Manual (ISM) was used as a guideline for identifying risks and controls when considering requirements and determining CDL security measures. The ISO/IEC 17799:2005 Information Technology – Security Techniques – Code of Practice for Information Security Management was also consulted in developing the CDL IT solution and security plan. As Figure 3 demonstrates, the CDL stand-alone network is physically separate from all other networks. The environment was later subjected to an independent, external security audit.

### Independent audit

The objectives of the independent audit were to review the CDL secure IT environment, and identify and describe the controls to ensure that they were being applied in compliance with the standards and processes identified by the PHRN stakeholders. The audit included a full review of the configuration, operations, and usage of the CDL infrastructure.

Among other things, the audit report provided an assessment of how the infrastructure was configured and used relative to the standards identified by the PHRN stakeholders and recommended changes to configuration and usage.

### Governance

A major challenge for all members of the PHRN has been to ensure that the collection, use and disclosure of personal information comply with applicable information privacy legislation. Compliance with legal requirements relating to privacy is essential but it is only one dimension of good governance. Equally important is the development of a strong culture of understanding and support for privacy goals and governance best practice.

Among the governance structures instituted by the PHRN are a Management Council overseeing the implementation of the national data linkage program, with sub-committees which provide advice and direction to Management Council members. These sub-committees include an Ethics, Privacy, and Consumer Engagement Advisory Group, an Operations Committee (providing technical advice) an Access Committee (providing advice on access, accreditation and eligibility); a Data Transfer Working Group and Proof of Concept Reference Group. Additional governance features of the PHRN include a strict reporting regime; a Privacy framework; an Information Governance framework; rigorous approvals processes for each research project; binding agreements related to data release, date confidentiality and security and Network-wide policies and guidelines.

### Software evaluation

A need to identify accurate, reliable, load-bearing (i.e. production capability) record linkage software was recognised in the very early stages of development. As a consequence, the CDL embarked on an evaluation of ten data linkage software packages to assess their suitability for inclusion in a large scale automated production environment [18,19]. The evaluation identified three potential candidate packages. These products were shortlisted for further testing during the Proof of Concept phase (POC; see below).

### PHRN proof of concept linkages

The primary aim of the PHRN Proof of Concept projects is to demonstrate the capability of the PHRN infrastructure to answer research questions of national importance, by conducting inter-state linkages [14]. The first PHRN Proof of Concept project examined in-hospital mortality and investigated issues of hospital safety and quality using inpatient and mortality information.

Initial data was provided to the CDL from NSW and WA. This comprised more than 25 million hospital and mortality records over a ten year period. Consistent with the Cross-Jurisdictional Model, data flows and linkage activity included the following:

•Transfer of hospital and mortality demographic information and jurisdictional linkage keys from custodians and linkage units in NSW and WA to the CDL

•Linkage of this data to create a national map

•Creation of project-specific linkage keys based on this map

•Transfer project-specific linkage keys back to the jurisdictions

•Transfer of the necessary clinical data from the jurisdictional custodians to the researcher

## Results and discussion

The CDL Cross-Jurisdictional Model was endorsed by the PHRN Management Council in 2010 [20]. A development and implementation programme based on that Model subsequently commenced (and is still on-going). The development programme includes the design and implementation of a large-scale automated production linkage system in which a national linkage map can be created and maintained over time as new datasets and updates to datasets become available.

### Strengths and weaknesses of the model

The Cross-Jurisdictional Model has a number of design strengths. Firstly, it implements the best practice separation model [16] to protect the privacy of individuals. Secondly, it adopts a “minimum data” principle in which participants are provided only with the minimum amount of information required to conduct their designated activity. Both of these elements are consistent with Australian government principles for data integration [21]. The Jurisdictional Linkage Units and encrypted versions of their jurisdictional linkage keys are integral to the process. They ensure that high quality linkages at both state and national level are maintained and that resources are used efficiently. The independence of Jurisdictional Linkage Units is also maintained under this Model, as is the proximal relationship between these Units and local data custodians. Finally, the Cross-Jurisdictional Model is designed to be extensible – datasets and/or linkage units can be added with minimum impact on the overall system.

Although the Model has been designed to maximise the protection of privacy, the additional data flows also introduce some operational restrictions. The obvious limitation is around the coordination of numerous “separated” elements before different datasets can be joined up. This process can be complex and requires careful consideration to avoid bottlenecks in the system. There are other limitations to the Model. For example, there is no flexibility in operations – roles of participants are defined from the start. Data flows are also likely to be slow and highly dependent on the capabilities and resourcing of Data Custodians. Processes may be difficult to speed up or streamline. System auditing is also more difficult under a “separated” Model, as it is difficult to trace the history of linked analytical data without good coordination and oversight.

This model was agreed to after extended consultation with the rest of the network. A consultation paper was presented to PHRN participants outlining proposed models and asking for feedback regarding particular options. The model was chosen based on a desire to find consensus amongst participants. Alternative models were proposed, including the CDL receiving data directly from state Data Custodians. Receiving data from linkage units allowed the CDL to leverage off the existing relationship between the data custodians and linkage units, and to utilise the jurisdictional linkage keys for quality assurance purposes.

### Operational governance and IT

The CDL has established a development programme which involves constructing effective matching methodologies around the agreed operational model. In addition to developing and demonstrating technical linkage capabilities, governance arrangements at the CDL were further developed and refined. The CDL has developed specific governance provisions around security and operations, risk management and privacy (including Privacy Impact Assessment). Ethics approval has been granted to operate the CDL cross-jurisdictional data linkage infrastructure.

A secure IT environment was established to meet the security standards developed as part of the PHRN Information Governance Framework for cross-jurisdictional data linkage. The environment was later subjected to an independent, external security audit as part of the threat and risk assessment process.

Overall the audit concluded that the CDL environment and systems were being managed in an efficient and reliable manner. Although no major deficiencies were observed, the report provided non-essential recommendations. All recommendations were addressed successfully. The independent audit review process has been included in the CDL Governance Plans which means that other audits will be required in the future if there are significant changes to the secure IT environment.

### Software evaluation

The software evaluation was successful in identifying appropriate software for production linkage. The software evaluation also resulted in the development of a unique, sharable methodology for data linkage software evaluation. The methodology incorporates the use of synthetic data and is both transparent and transportable [22]. The knowledge and expertise developed through the evaluation was shared with the wider PHRN to assist their developments.

### PHRN proof of concept linkages

The cross-jurisdictional data linkage capabilities of the CDL have been demonstrated through involvement in the PHRN Proof of Concept Collaboration projects. Using its data linkage capabilities, the CDL linked both NSW and WA data as new and compared these results to those achieved by the WA Data Linkage Branch (WADLB) and the NSW CHeReL. The jurisdictional linkage keys supplied by the linkage units in NSW and WA were purposely not used during the linkage process, but were used solely to measure linkage quality once the CDL had completed its linkages. By comparing the CDL links with those of the jurisdictions, the CDL was able to evaluate its ability to link very large dataset to a high quality in a short period of time. The results for all linkages were exceptionally high. In total, 99.2% of links found by the CDL were correct, and 96.8% of all links were found. The CDL was successful at closely replicating jurisdictional links in a short time span. The CDL obtained an overall linkage accuracy measure (F-measure) of 0.99 for WA data, and 0.97 for NSW data. Both results were very high. The lower linkage quality obtained for NSW data could be attributed to poorer data quality.

Additional projects utilising cross-jurisdictional linkage infrastructure are in train. These include an exploration of the burden and cost of health care due to injury (which utilises state morbidity, emergency and mortality datasets) and an investigation into the role of perinatal factors in the developmental and educational outcomes of Australian children, (using state level birth and perinatal datasets and the Australian Early Development Index, a national collection on young children’s development [23]). The range of possible research projects which can use cross jurisdictional linked data is large and diverse and will have the capacity to improve government policy and planning. The possibility for data linkage research looks set to be restricted only by imagination.

### Progress

As results show, the CDL has met its objective of “establishing a secure and efficient data linkage system to facilitate linkage between jurisdictional datasets” [14]. The CDL has established a secure IT environment, instituted strong governance arrangements and implemented a unique cross-jurisdictional operational model. As evidenced by Proof of Concept linkage results, the CDL has also developed the technical capability to undertake large-scale data linkage and produce high-quality linkage output.

### Current developments

The CDL is currently continuing with the development of a full production linkage system. In the past, production linkage systems have been limited by their inability to handle increasingly large datasets. The major reason for this poor scalability is the exponential growth in the number of possible matches as so-called “master datasets” extend. To address this and ensure sustainability of national infrastructure, the CDL has designed an efficient and sustainable component-based production linkage system. The system has been designed to securely link event data based on probabilistic matching of demographic information. A new grouping methodology has been implemented that operates at record-pair level. The system has the functionality to support changes in records and datasets over time. Additionally, the linkage system provides functionality to support its own administration by operational staff.

The issues in implementing cross jurisdictional linkage are not only technical. There are also significant challenges around management and governance, engagement with stakeholders, and working in a federated environment with differing legislation. The researchers working with cross jurisdictional linked data also face challenges around merging data from different states and working with different collection methodologies and variable definitions.

### Future directions

Data linkage in Australia is an evolving space. At the same time as the PHRN and CDL were developing, a number of Commonwealth government agencies came together to establish a set of guiding principles for data integration involving Commonwealth data [21]. Governance and institutional arrangements for Commonwealth data integration projects have now also been articulated and an accreditation process has recently been put in place.

With safeguards in place, it should be possible to adapt the existing CDL Cross-Jurisdictional Model to accommodate the linkage of State-based datasets to Commonwealth-held data. The resulting infrastructure would provide a resource which can be used to create epidemiological and management information that can be used to investigate and model interactions within a complex, federated Australian health system. Data linkage at this scale would significantly improve Australia’s capacity to carry out population health research at a truly national level.

## Conclusion

Governments and universities in Australia understand that linked administration data can provide an unparalleled resource for the monitoring and evaluation of services. However, for a number of reasons, these data have not previously been readily available to researchers.

The infrastructure established by the CDL presents a major opportunity to exploit administrative collections and improve the quality of population research data across Australia, with the consequential benefits of improved health and wellbeing of Australians.

## Pre-publication history

The pre-publication history for this paper can be accessed here:

http://www.biomedcentral.com/1472-6963/12/480/prepub
